# Supplementary material for: Long‐Lived Photoluminescence of Photostable One‐Dimensional Picoperovskites
Source: Small Sci. 2026 Feb 8;6(2):e202500427. doi: 10.1002/smsc.202500427 (PMC12884787; doi:10.1002/smsc.202500427)
Supplement: Supplementary file 1 — Supplementary Material [file SMSC-6-e202500427-s001.pdf]

Supplementary Information

PL spectra were obtained under continuous laser excitation with an integration time of 1s. Each data point corresponds to the integrated PL spectra for the referred time after beginning the illumination. The degradation curve for a bulk sample under excitation power of 50 µW shows an exponential decay, same for a pico-perovskite sample under excitation power of 32 µW. The decrease of intensity for the bulk sample is slower than for the pico sample, possibly due to oxidation effects, leaving deeper layers intact or slowing the process. For an excitation power of 10 µW the behavior of the picoperovskite sample drastically changes. No clean exponential decay can be identified, instead the trace consists of different regions with increasing and decreasing intensity, which can have multiple reasons. We assume a combination of laser drift and position change. The excitation can locally increase the temperature, fundamentally rotating the inorganic octahedra of the perovskite tissue, not excluding other temperature related effects.

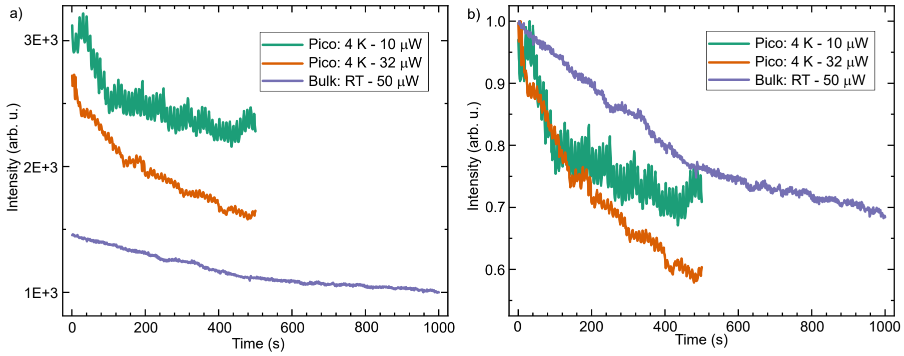

Figure 1: Amplitudes obtained by fitting multiple PL spectra with 1 s integration time under constant laser exposition performed on sample 1 for two different laser powers and a bulk sample for comparison. a) absolute, b) normalized values.

Halide perovskites $CsPbI_3$ were analysed using Malvern Panalytical's Empyrean diffractometer to perform X-ray powder diffraction (XRD). Samples of $CsPbI_3$ were measured for 40 minutes each with the range of angles 2θ=10-70° to account for not only the peaks for $CsPbI_3$, but also for the silicon holder and base so its peaks can be subtracted from the data.

Lattice parameter	Expected (Å)	Observed (Å)	Observed error (Å)	Standard error
a	10	10	0	6
b	4	4	0	8
C	17	17	0	5

Table 1: Lattice parameters of the measured CsPbI3 compared to well-known lattice parameters (from I. Chung, et al. and D. B. Straus, et al.)

Figure 2 shows the sample's XRD spectra with the range $2\theta \approx$20-40° as it is where the most visible peaks occur for $CsPbI_3$. The wide peaks of $2\theta \approx$19.9°, 21.9°,24.0°,30.4°, 35.0° are from the background (e.g. silicon). The most intense γ-$CsPbI_3$ peaks at 28.6° and 29.0° can be barely seen above background, meaning most of the sample is δ-$CsPbI_3$. This is because of phase change of γ-$CsPbI_3$ back to δ-$CsPbI_3$ at room temperature. All the $CsPbI_3$ peaks are much stronger and clearly defined. It is worth noting that upon high temperature encapsulation the structure returns to γ-$CsPbI_3$ phase and encapsulated picoperovskites retain γ phase. The lattice parameters of the $CsPbI_3$ samples were calculated after refining the data. The following table gives the observed and expected lattice parameters and the number of standard deviations between the observed and expected using the difference between the expected and observed over the observed error.

Table 1 shows that the expected and observed lattice parameters are very close to each other, so the first and second perovskites are confirmed yellow phase δ-$CsPbI_3$. The standard error is high because the observed error has been underestimated as it may not account for the random errors in the diffractometer's position and count measurements.

For the absorption spectra shown in Fig.5 and the source powders were dissolved in isopropanol and measured under constant stirring by a magnetic stirrer, as the dispersion would otherwise drop to the bottom of the cuvette. The scattering offset was approximated by a 4th order polynomial fit to the data in the non-absorbing region below (above) 1,774 eV (700 nm) and substracted from all curves. Furthermore the absorption spectrum of isopropanol was substracted from all curves.

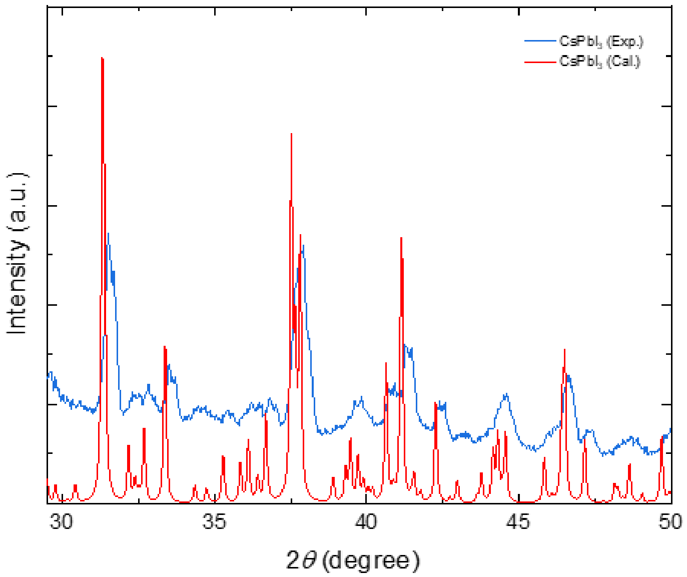

Figure 2: Composite XRD spectra of experimental and calculated $CsPbI_3$

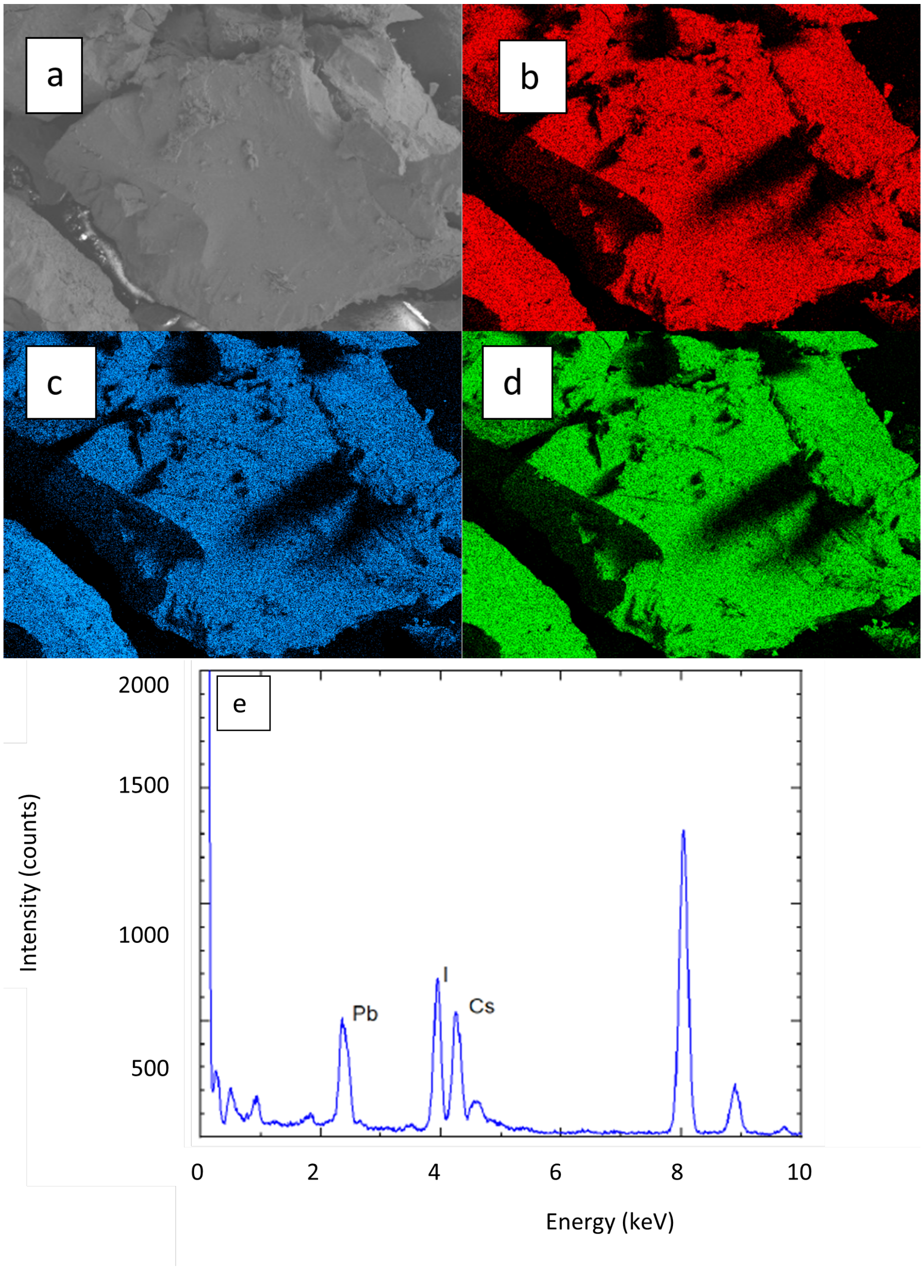

Figure 3: (a) SEM micrograph of CsPbI3 particles, (b-d) EDS elemental maps of the area in (a), (e) a typical EDS spectrum with Cs, Pb, and I labeled, used to produce the maps. Peaks at around 8 and 9 are from a copper grid. SEM micrograph.

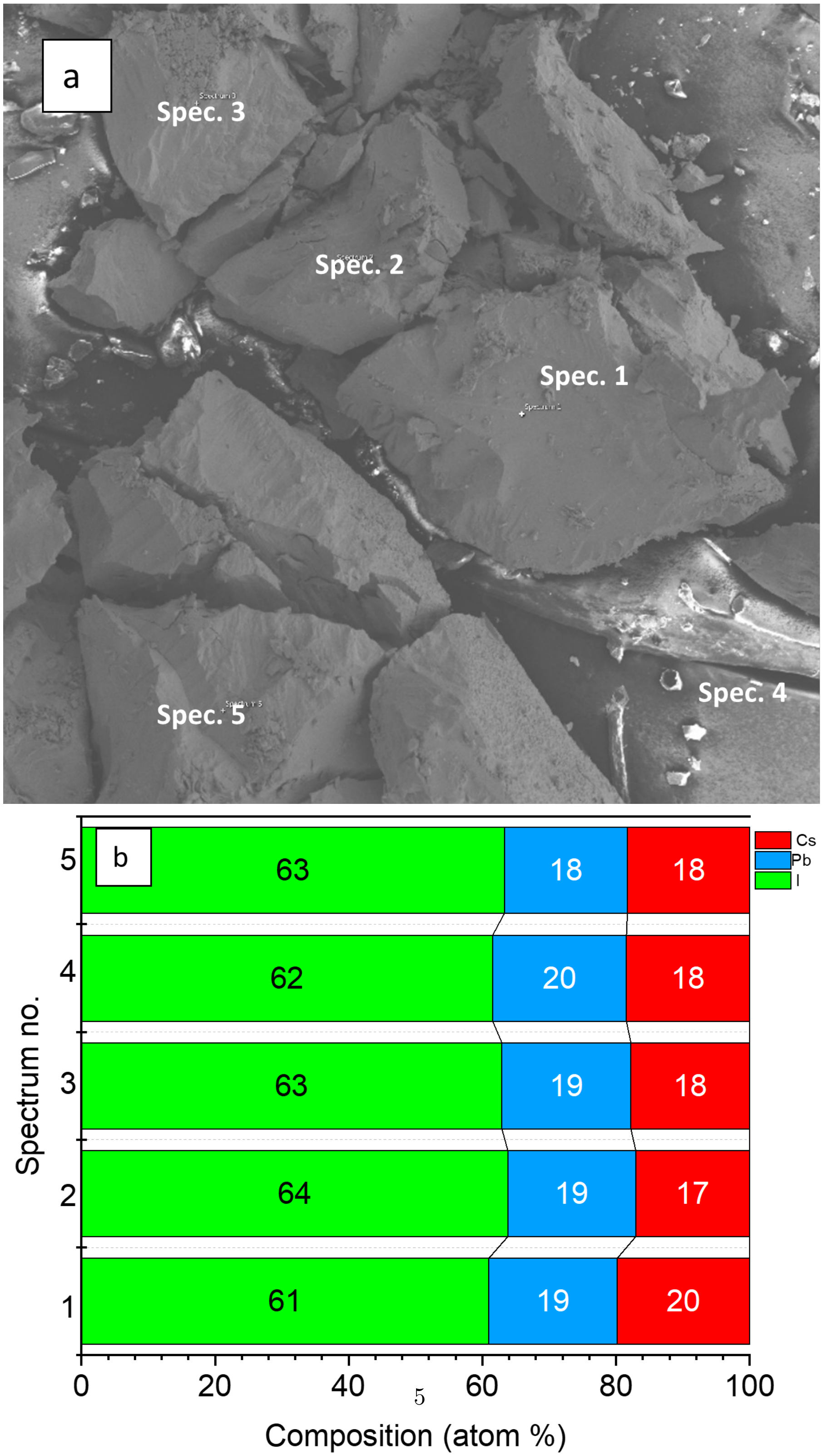

Figure 4: (a) SEM micrograph of few CsPbI3 particles and (b) stacked columns representing their relative chemical composition of Cs (red), I (green), and Pb (blue) in atomic%.

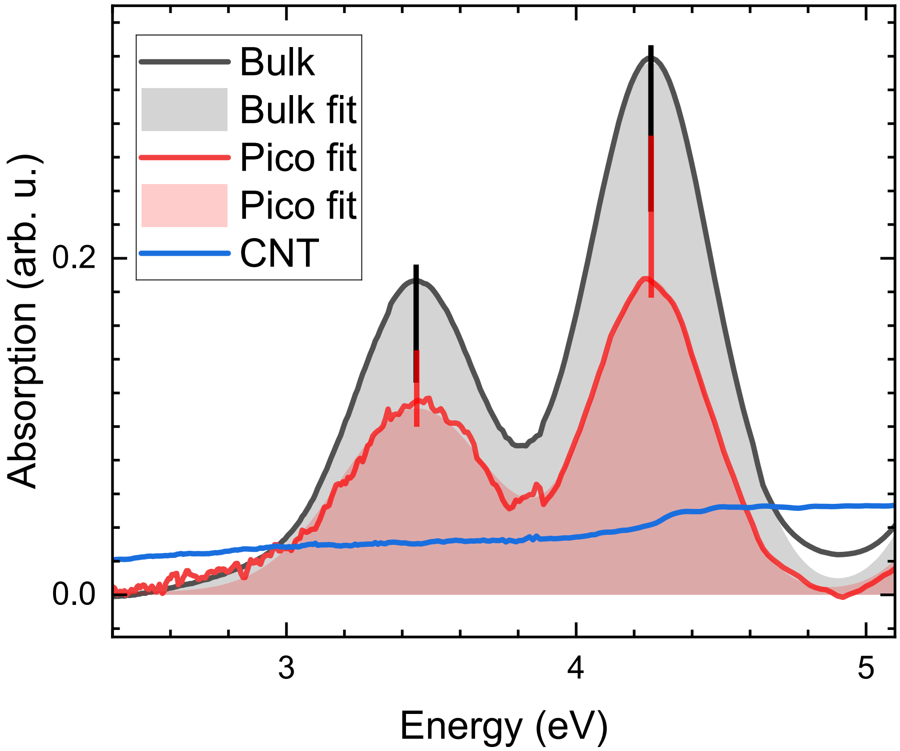

Figure 5: Absorption spectra of a bulk (black line), a pico-perovskite (red) sample and the unfilled CNTs (blue). The transparent area is the cumulative fit for the bulk (black) and the pico (red) perovskite. The vertical lines show the peak positions for the two interband transitions.

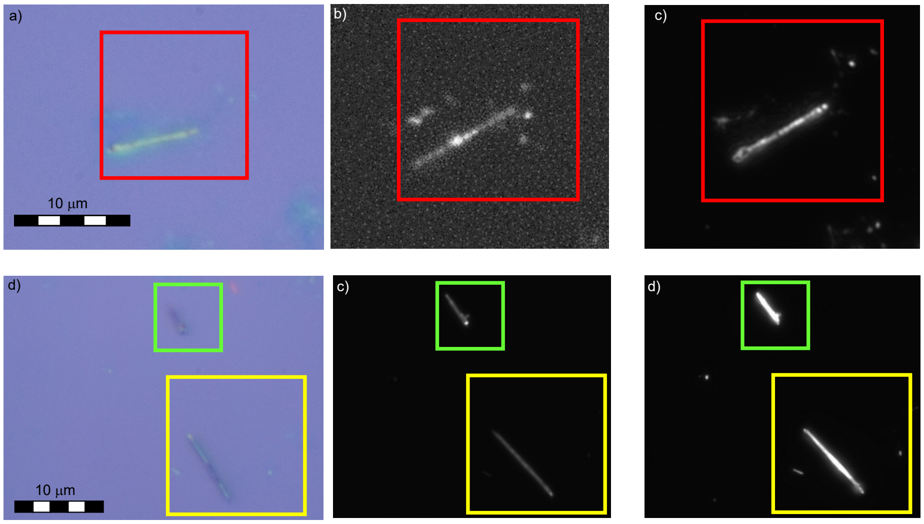

Figure 6: a), b), c) Optical, Fluorescence, Dark field image of sample 1. d), e), f) Optical, Fluorescence, Dark field image of sample 2 and 3.

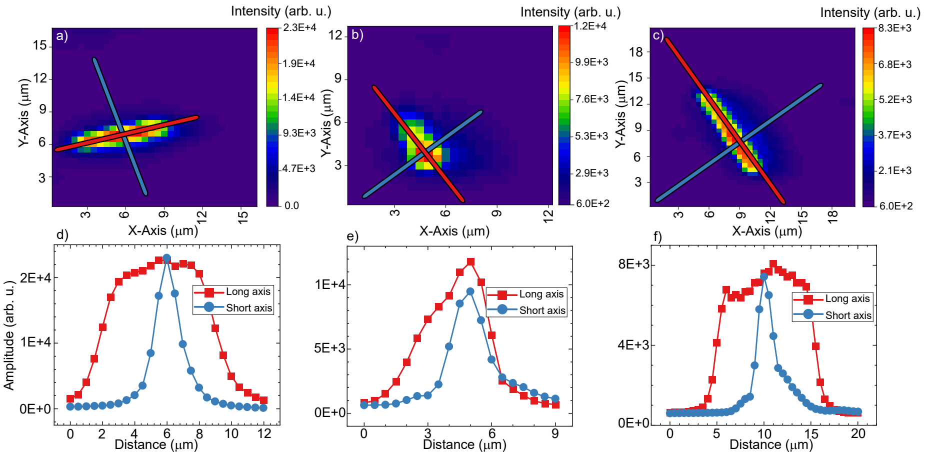

Figure 7: a), b), c) False color map of PL intensity extracted from a scan of the area marked by the boxes in **Figure 6** on sample 1, 2 and 3. d), e), f) Linescans obtained from the maps shown in a), b) and c) noted by the red and blue colored bars.

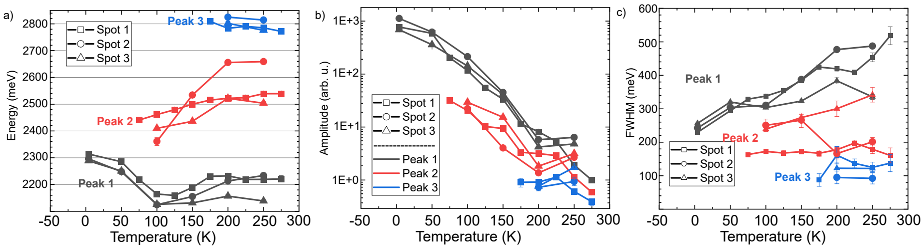

Figure 8: a) Energy, b) Amplitude and c) FWHM of a PL temperature series for all 3 samples.

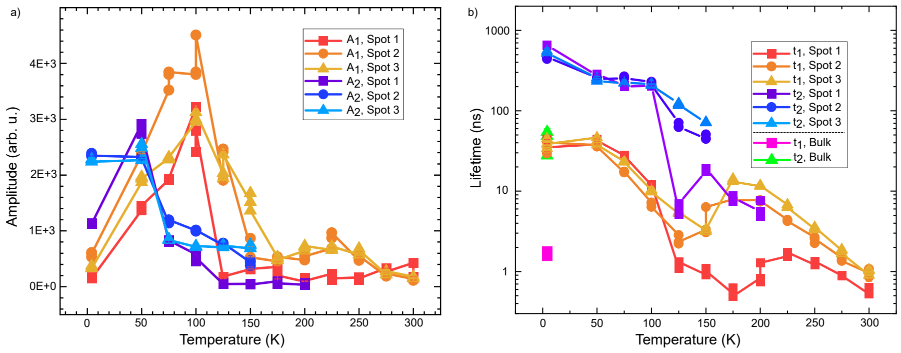

Figure 9: a) Amplitude and b) Lifetime from TRPL temperature series for all 3 different samples and the results from 1 Bulk sample at 4K.
